# Role of connexin 43 in odontoblastic differentiation and structural maintenance in pulp damage repair

**DOI:** 10.1038/s41368-020-00105-1

**Published:** 2021-01-08

**Authors:** Jiaxin Yin, Jue Xu, Ran Cheng, Meiying Shao, Yuandong Qin, Hui Yang, Tao Hu

**Affiliations:** 1 State Key Laboratory of Oral Diseases & National Clinical Research Center for Oral Diseases & West China Hospital of Stomatology, Sichuan University, Chengdu, China; 2 Department of Endodontics, School of Stomatology, Capital Medical University, Beijing, China; 3 West China School of Public Health and West China Fourth Hospital, Sichuan University, Chengdu, China

**Keywords:** Pulpitis, Stem-cell differentiation, Dental caries

## Abstract

Dental pulp can initiate its damage repair after an injury of the pulp–dentin complex by rearrangement of odontoblasts and formation of newly differentiated odontoblast-like cells. Connexin 43 (Cx43) is one of the gap junction proteins that participates in multiple tissue repair processes. However, the role of Cx43 in the repair of the dental pulp remains unclear. This study aimed to determine the function of Cx43 in the odontoblast arrangement patterns and odontoblastic differentiation. Human teeth for in vitro experiments were acquired, and a pulp injury model in Sprague-Dawley rats was used for in vivo analysis. The odontoblast arrangement pattern and the expression of Cx43 and dentin sialophosphoprotein (DSPP) were assessed. To investigate the function of Cx43 in odontoblastic differentiation, we overexpressed or inhibited Cx43. The results indicated that polarized odontoblasts were arranged along the pulp–dentin interface and had high levels of Cx43 expression in the healthy teeth; however, the odontoblast arrangement pattern was slightly changed concomitant to an increase in the Cx43 expression in the carious teeth. Regularly arranged odontoblast-like cells had high levels of the Cx43 expression during the formation of mature dentin, but the odontoblast-like cells were not regularly arranged beneath immature osteodentin in the pulp injury models. Subsequent in vitro experiments demonstrated that Cx43 is upregulated during odontoblastic differentiation of the dental pulp cells, and inhibition or overexpression of *Cx43* influence the odontoblastic differentiation. Thus, Cx43 may be involved in the maintenance of odontoblast arrangement patterns, and influence the pulp repair outcomes by the regulation of odontoblastic differentiation.

## Introduction

The pulp–dentin complex is a unique tissue that can respond to external stimuli and repair itself because the monolayer odontoblasts surrounding dental pulp can secrete mineralized matrix (tertiary dentin) to defend against injury.^1,2^ Under moderate injury, odontoblasts can secrete reactionary dentin (a type of tertiary dentin) to isolate the injury and protect the pulp.^1,3^ When the pulp is stimulated by severe injury, odontoblasts are destroyed, and the progenitor cells in the dental pulp, including dental pulp stem cells,^4,5^ Höhl cells,^6^ dental pulp pericytes,^7^ or smooth muscle actin-positive (SMA^+^) progenitors,^8^ are recruited to the injury site and differentiate into odontoblast-like cells that secrete reparative dentin (another type of tertiary dentin) to eventually repair all or part of the damaged areas.^1,3,9–11^ In some cases, osteodentin is formed from differentiated dental pulp cells (DPCs) surrounded by extracellular mineralized matrix. Osteodentin is characterized by lacunar bone-like tissue and is histologically considered immature mineralized tissue, which is a type of reparative dentin.^12,13^ However, the specific regulatory mechanism of the formation of reparative dentin is unclear.

Odontoblasts are highly polarized cells perpendicular to the inner surface of dentin.^14–16^ Cell polarity endows the cells with variable structure and function, which is required for the migration, development, and intercellular communication of the majority of the human cells.^17^ Cell polarity and palisade structure of odontoblasts are the basis for the formation of the odontoblast arrangement patterns and tubular dentin.^18^ Therefore, the arrangement pattern of odontoblasts and maintenance of the odontoblast structure play fundamental roles in primary dentin and in the formation of tertiary dentin during damage repair.

Cell arrangement relies on the cell–cell junctions. Gap junction, one of the cell junctions, is considered important for the maintenance of homeostasis of the internal environment and is involved in the directional differentiation of the target cells.^19–21^ Gap junctions are composed of proteins encoded by the connexin gene family; connexin 43 (Cx43) is the most common and abundant connexin. Cx43 expression and phosphorylation levels influence the function of the gap junctions.^22^ A previous study demonstrated that Cx43 is involved in the maintenance of the tissue structure in several human organs.^23^ Cx43 may be essential for the formation and maintenance of cell polarity.^24^ Mutation of the human Cx43-encoding gene *GJA1* leads to oculodentodigital dysplasia, an autosomal dominant genetic disease characterized by craniofacial anomalies involving teeth and skull.^25^ Hashida et al. demonstrated that Cx43-mediated intercellular gap junctions are involved in the differentiation of osteoblasts, and silencing *GJA1* can directly cause a decrease in the mineralization of osteoblasts.^26^ Moreover, *GJA1* knockout in zebrafish leads to abnormal skeletal development and short fin phenotype.^27^ Cx43 was also shown to be associated with odontoblastic differentiation.^28^ However, the role of Cx43 in the formation of reparative dentin and maintenance of odontoblast arrangement is poorly understood.

In this study, we initially investigated the normal odontoblast arrangement and Cx43 expression patterns in human, rat, and dog. A pulp damage repair rat model and an in vitro model were used to investigate the relationships between Cx43 and the odontoblast arrangement pattern and odontoblastic differentiation.

## Results

### Odontoblast arrangement and Cx43 expression pattern in healthy teeth

**Fig. 1:**
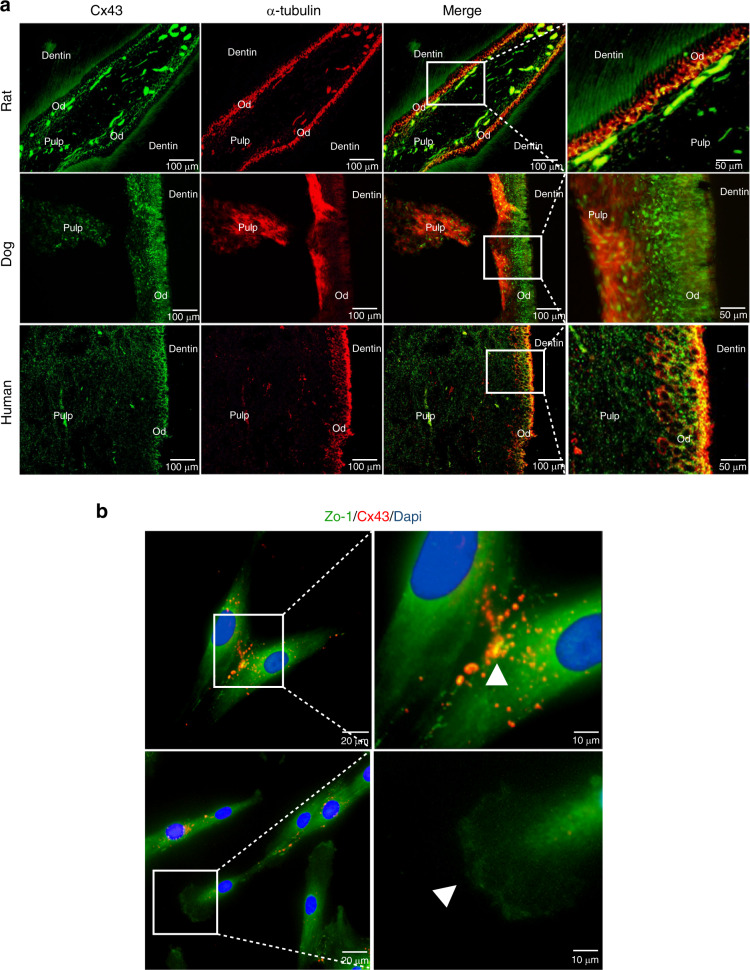
Odontoblast arrangement structure and Cx43 expression in the dental pulp. **a** Cx43 and α-tubulin immunofluorescence staining in rat, beagle, and human healthy dental pulp tissue. **b** Immunofluorescence staining of Cx43 and ZO-1 in dental pulp cells cultured in vitro. Od, odontoblasts

The palisade-like structure was detected in the odontoblastic layer in the healthy teeth of rat, beagle dog, and human (Fig. 1a). Polarized odontoblasts have higher expression of Cx43 compared with that in the pulp tissue (Fig. 1a).

Coexpression of a tight junction protein zonula occludens-1 (ZO-1) and Cx43 was detected in hDPCs in vitro (Fig. 1b); however, the expression of Cx43 was barely detectable in the ZO-1-enriched cytoplasmic pseudopodia (Fig. 1b).

### Odontoblast arrangement and Cx43 expression in the carious human teeth

**Fig. 2:**
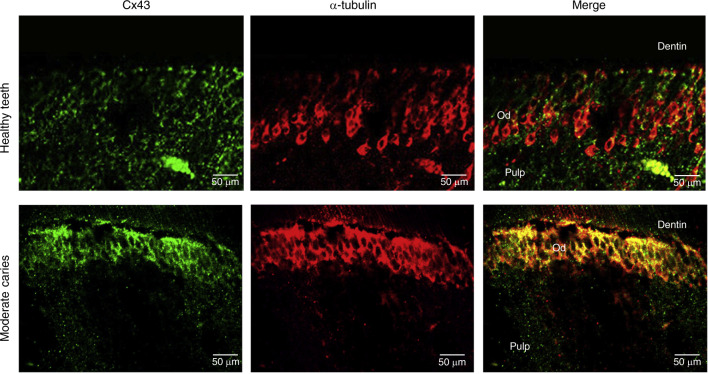
Cx43 expression in the pulp tissue stimulated by caries. Cx43 (green) and α-tubulin (red) immunofluorescence staining of the odontoblast layer in teeth with moderate caries and healthy controls. The expression of Cx43 is slightly upregulated in caries teeth. Od, odontoblasts

Compared to the healthy human teeth (Fig. 2), the odontoblast arrangement pattern was slightly changed concomitant to an increase in the expression level of Cx43 in teeth with moderate caries (Fig. 2). Although the arrangement pattern was slightly changed, the odontoblast layer was detected, and the arrangement of the cells was organized. These data suggest that the cells might be original odontoblasts, and their function was stimulated.

### Odontoblast arrangement and Cx43 expression in the rat teeth with inflammatory or necrotic dental pulp

**Fig. 3:**
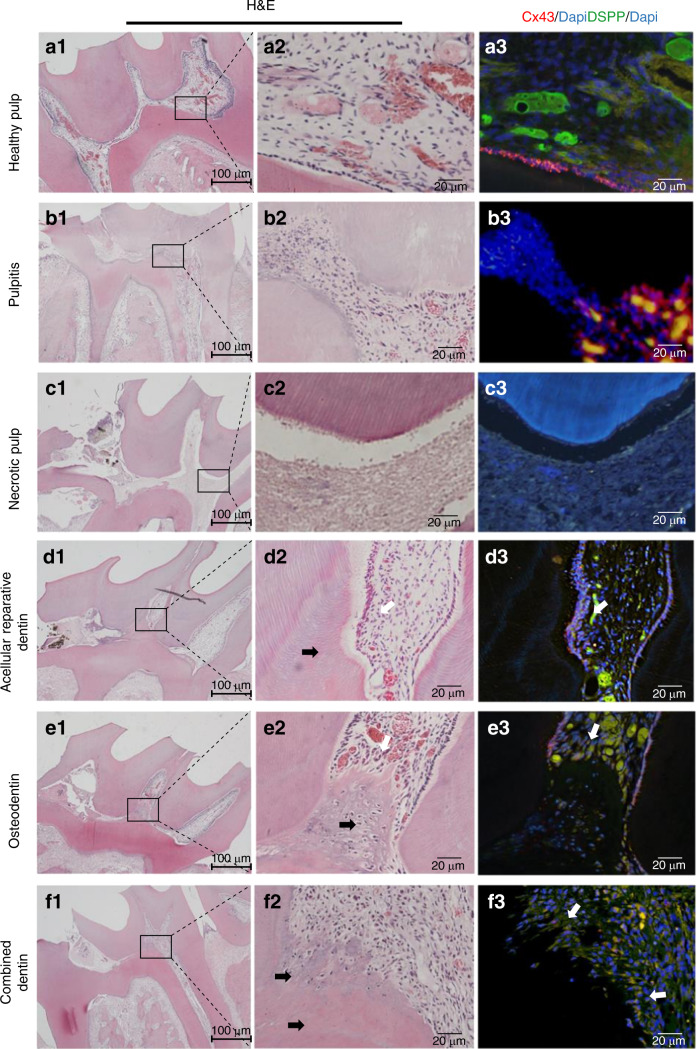
Cx43 expression and different dental pulp repair patterns in vivo. H&E and immunofluorescence staining in normal (**a1**–**a3**), inflammatory (**b1**–**b3**), necrotic (**c1**–**c3**), and reparative dentin pulp tissues (**d1**–**f3**) in rats. Regularly arranged odontoblast-like cells are present beneath acellular reparative dentin (**d1**–**d3**); however, these cells or structure are not detected beneath osteodentin (**e1**–**e3**)

In the control group, odontoblasts were arranged in a regular pattern surrounding the pulp tissue (Fig. 3a1–a3), and Cx43 (labeled by red fluorescence) and DSPP (labeled by green fluorescence) were coexpressed in the odontoblast layer.

In the pulpitis group (inflammatory pulp), the distinct odontoblastic layer apparently disappeared (Fig. 3b1–b3). Additionally, the formation of reactionary or reparative dentin was barely detectable. Furthermore, odontoblasts were completely absent as shown in the left part of Fig. 3b2. Immunofluorescence staining demonstrated the disarranged nuclei of odontoblasts (Fig. 3b3).

The odontoblastic layer was also completely absent in the necrotic dental pulp compared with that in the control group. Cellular structure and Cx43/DSPP expression beneath the dentin were absent (Fig. 3c1–c3).

### Odontoblast arrangement and Cx43 expression in different pulp repair models

In our damage repair animal model, three different repair models of the newly formed dentin were detected. To investigate the characteristics of the reparative dentin, we assessed odontoblast arrangement and determined the expression pattern of Cx43 in odontoblasts and pulp tissue.

#### Model 1: Formation of acellular reparative dentin

A large area of acellular reparative dentin (tubular or atubular dentin), most of which was tubular dentin, was formed with relatively regularly arranged odontoblast-like cells on the inner surface (Fig. 3d1–d3). Immunofluorescence staining demonstrated elevated expression of Cx43 in newly formed odontoblast-like cells compared to that in other regions of the pulp tissue (Fig. 3d1–d3).

#### Model 2: Formation of osteodentin

Osteodentin was formed by newly differentiated odontoblast-like cells adjacent to the injured area (Fig. 3e1, e2). Cell bodies were scattered in osteodentin and were encapsulated in it (Fig. 3e2). Moreover, there was no boundary or regular margin of osteodentin. The pulp tissue was slightly hyperemic, and regularly arranged odontoblast-like cells were not detected beneath osteodentin (Fig. 3e1–e3).

Distinct Cx43 expression was not observed in the cells beneath osteodentin. Osteodentin was different from the tubular dentin described above and was lacunar without tubular structures. Cell polarization or palisade-like structures were not detected.

#### Model 3: Formation of acellular reparative dentin and osteodentin

Coexistence of acellular reparative dentin and osteodentin was detected in some instances. Acellular reparative dentin was formed adjacent to the damaged region (Fig. 3f1), and disorganized tubular dentin or atubular dentin was also detected (Fig. 3f1, f2). Osteodentin was detected between acellular reparative dentin and dental pulp (Fig. 3f2). Regularly aligned odontoblast-like cells were barely detectable beneath osteodentin (Fig. 3f2). Immunofluorescence staining showed lower expression level of Cx43 in the cells beneath osteodentin. These data suggest that Cx43 has a certain regulatory effect on the odontoblastic differentiation of DPCs and arrangement pattern of odontoblast-like cells to further influence the dentin formation (Fig. 3f1–f3).

### Cx43 expression is associated with odontoblastic differentiation

**Fig. 4:**
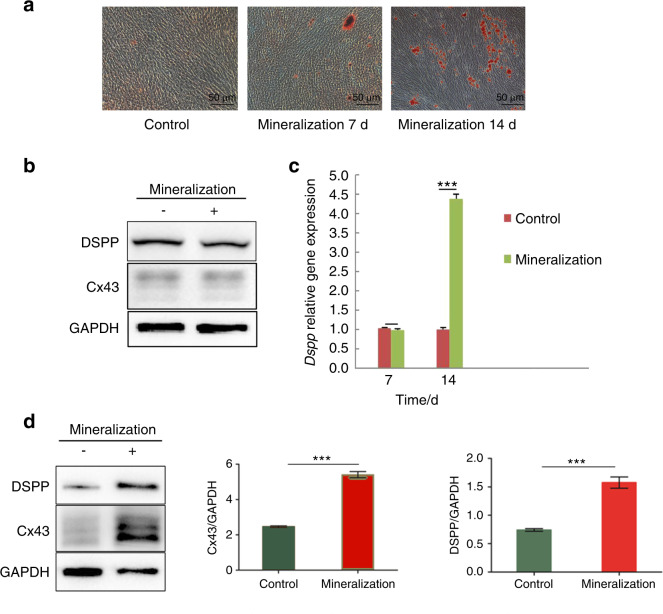
Cx43 expression and odontoblastic differentiation in vitro. **a** After differentiation induction, dental pulp cells were stained with Alizarin red. **b** Western blot was used to detect the protein expression level of DSPP and Cx43 after 7-day induction, and **d** 14-day induction. **c** Real-time PCR was used to assess the *DSPP* gene expression

Culture of hDPCs in mineralization solution for 7 and 14 days induced an increase in the number of mineralized nodules in a time-dependent manner (Fig. 4a). However, the expression of DSPP and Cx43 proteins (Fig. 4b), and *DSPP* gene (Fig. 4c) was the same as in control groups after 7-day mineralization induction. The expression of *DSPP* gene and the proteins increased only after 14-day induction (Figs. 4c, d). The expression patterns of Cx43 and DSPP have a similar trend during the odontoblastic differentiation of hDPCs.

### Silencing or overexpression of Cx43-encoding gene *GJA1* is closely associated with odontoblastic differentiation of hDPCs

**Fig. 5:**
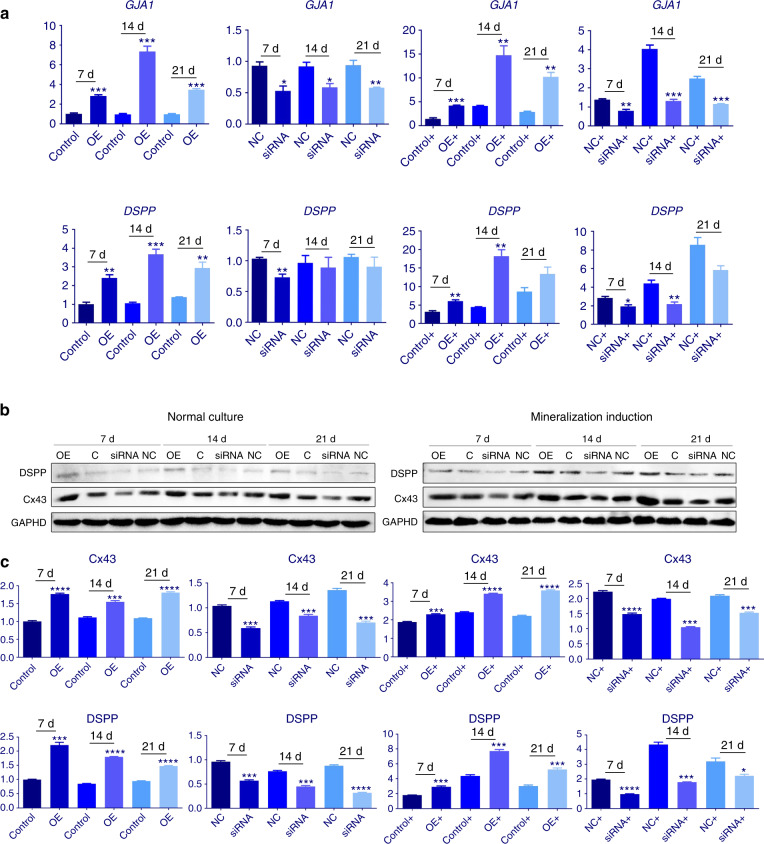
Odontoblastic differentiation of dental pulp cells with overexpressed or inhibited Cx43 in vitro. **a** Gene expression of *GJA1* and DSPP, and **b, c** protein expression of DSPP and Cx43 at 7, 14, and 21 days were detected after inhibition or overexpression of the Cx43-encoding *GJA1* gene. OE, over-expression group; control, OE null group; siRNA, gene silencing group; NC, siRNA null group; +, mineralization induction

To investigate whether the expression level of Cx43 influences the odontoblastic differentiation of hDPCs, *GJA1* was silenced or overexpressed; the results confirmed the inhibition and overexpression of *GJA1* (Fig. 5a). The *GJA1* silencing and control groups had similar *DSPP* transcription levels when mineralization was not induced. After mineralization induction in the culture, the *DSPP* gene (Fig. 5a) and protein (Fig. 5b, c) levels in the *GJA1* silencing group were lower than those in the control group. Notably, with or without mineralization induction for 7 or 14 days, the *DSPP* transcript levels were higher in the *GJA1* overexpression group than that in the control group (Fig. 5a). Therefore, Cx43 overexpression resulted in upregulation of DSPP under mineralization induction and normal culture conditions, but Cx43 inhibition led to DSPP downregulation only under mineralization induction. Thus, the results indicate that Cx43 may play an indispensable role in the odontoblastic differentiation of hDPCs. We could also find that the Cx43 in the overexpression and silencing groups was stably expressed after 7, 14, and 21 days culture (Fig. 5c).

## Discussion

Tertiary dentin can be formed in specific loci at the pulp–dentin interface in response to external stimuli. The structure of tertiary dentine can vary from a regular tubular matrix to a very dystrophic pattern; the tubular matrix possibly contains entrapped cells.^1^ The process of reparative dentin formation is completely different from that of reactionary dentin.^29^ Reactionary dentin is formed by the original postmitotic odontoblasts, which are responsible for primary dentinogenesis, and the reparative dentin is secreted by a new generation of odontoblast-like cells, which are differentiated from pulp precursor cells.^30^ In the case of severe damage, progenitor cells in the dental pulp are recruited to the injury site and differentiate into odontoblast-like cells to repair the tissue after the death of original odontoblasts.^30–34^ The source of odontoblast-like cells has not been determined. A generally accepted theory suggests that these cells originate from the dental pulp stem cells, which are also the progenitor cells of odontoblasts.^35^ Another theory suggests that Höhl cells may serve as progenitors to form odontoblast-like cells during tertiary dentinogenesis. These cells are of mesenchymal origin and are referred to as preodontoblast progenitor cells or simply as fibroblasts. In the case of an injury or a decline of primary odontoblasts, Höhl cells may differentiate into odontoblast-like cells and form reactionary dentin.^6^ DPCs have stem cell characteristics and odontoblastic differentiation potential;^36–38^ hence, we directly used DPCs in our in vitro study. Many studies have attempted to determine the mechanism of tertiary dentin formation; however, the mechanism is poorly understood. Thus, determination of the potential mechanism of initiation of odontoblastic differentiation and formation of a specific arrangement pattern is important for dental pulp repair.

Human odontoblasts are highly polarized cells with a palisade-like structure, which provides for dentin secretion and perception of the stimuli.^39^ However, it is unclear whether the odontoblast arrangement patterns play a role in the odontoblast function. The results of the present study indicate that the odontoblast palisade-like structure is common in the mammalian dental pulp tissues. Regularly arranged cells along the edges of the reactionary dentin and reparative dentin were detected; however, these cells were not detected in osteodentin. This result suggests that the odontoblast arrangement pattern may directly influence the function of odontoblast-like cells and subsequent secretion of the dentin matrix.

Cx43 is the most ubiquitously expressed connexin detected in various organs, tissues, and cells.^40^ Couve et al. found that Cx43 expression in odontoblasts beneath the carious lesions is lower than that in the healthy pulp tissues; however, no explanation was given for this phenomenon.^41^ Farahani et al. found that Cx43 expression is elevated in odontoblasts under caries.^42^ Muramatsu et al. found that Cx43 expression in the dental pulp tissues is gradually decreased with age and suggested that Cx43 expression is related to the activity of the dental pulp tissue.^43^ Additionally, Cx43 is downregulated during apoptosis.^44^ These results suggest that Cx43 expression is closely related to the odontoblast function. In our study, Cx43 protein expression was upregulated in odontoblasts in caries. These odontoblasts maintained the cell polarity with a palisade arrangement pattern (Fig. 2) but had elevated expression of Cx43. We suggest that these cells are the original odontoblasts with activated function. Since Cx43 is involved in maintenance of cellular polarity,^24,45,46^ Cx43 may interact with other junction proteins, such as ZO-1, to form a connection complex or to modulate the cell junctions^47^ to assist the cells in the maintenance of the integrity of the palisade barrier structure.^48^ The data of in vitro immunofluorescence staining demonstrated that Cx43 is mainly expressed at the cell–cell interface and is coexpressed with ZO-1; thus, we suggest that Cx43 participates in the maintenance of the odontoblast structure and in tertiary dentin formation. Our results obtained in the pulp damage repair models confirm this hypothesis. Regularly aligned odontoblast-like cells have elevated Cx43 expression associated with the formation of mature acellular reparative dentin. However, immature osteodentin is formed without typical odontoblastic arrangement pattern suggesting that Cx43 may be involved in the maintenance of the arrangement pattern and regulation of the odontoblastic differentiation of newly formed ondontoblast-like cells. These results suggest that Cx43 may be associated with the selection of a pulp damage repair mode.

To verify our hypothesis, an in vitro study was performed, and the data indicate that Cx43 gene transcription and protein expression are upregulated concomitant to an increase in the DSPP expression during odontoblastic differentiation. DSPP is an important and routinely used marker of odontoblastic differentiation with essential biological functions in dentinogenesis.^49,50^ Therefore, Cx43 may participate in odontoblastic differentiation and matrix mineralization. Cx43 is expressed at a high level adjacent to the mineralized nodules in the cultured DPCs compared with that in other regions.^51^ Cx43 silencing results in reduced ALP activity of DPCs.^52^ In our study, Cx43 overexpression promoted DSPP expression with or without odontoblastic induction. Cx43 inhibition induced a decreased DSPP expression compared with that in the control group after odontoblastic induction. Since DSPP is considered an important marker for odontoblastic differentiation,^8,37,53^ and we found same expression trend of DSPP and Cx43 both in protein and gene levels, we suggest that Cx43 is an indispensable protein for odontoblastic differentiation of hDPC.

From this study, we found Cx43 is involved in odontoblastic differentiation and is closely associated with the structural maintenance of odontoblasts and newly differentiated odontoblast-like cells. Differences in the expression level of Cx43 may result in different pulp damage repair modes. However, specific role and mechanism of Cx43 require further studies.

## Materials and methods

### Sample preparation

This study was approved by the ethics committee of the West China Hospital of Stomatology, Sichuan University. Impacted human third molars were collected from adults (between 18 and 25 years of age), and an informed consent was obtained from all participants. Healthy tooth specimens from beagle dogs and Sprague-Dawley (SD) rats were acquired. The tooth samples were fixed in 4% paraformaldehyde for two weeks and decalcified with 0.5 mol·L^−1^ ethylenediaminetetraacetic acid (EDTA) solution for 6 months before paraffin embedding.

### Cx43 expression assay

Demineralized human and dog tooth samples were cryoprotected successively in 15% and 30% sucrose in phosphate-buffered saline for 24 h and frozen in tissue-freezing medium (Tissue-Tek, Sakura Finetek, Torrance, CA, USA); 20-µm-thick dental pulp sections were cut using a cryostat (Leica CM-1900, Nussloch, Germany) at −20 °C. The sections were rehydrated in phosphate-buffered saline and incubated for 1 h in blocking solution containing 5% bovine serum albumin and 0.25% Triton X-100. Primary antibodies, including rabbit anti-Cx43 (1:1 000; Abcam, Cambridge, MA, USA) and mouse anti-α-tubulin (1:1 000; Abcam, Cambridge, MA, USA) were diluted in blocking solution and incubated overnight at 4 °C. Secondary antibodies were incubated for 1 h at room temperature. All tissue sections were mounted using the Fluomount mounting medium (Dako Industries, Carpenteria, CA, USA). The immunolabeled sections of the dental pulp were imaged using a confocal microscope (Nikon C1 plus, Nikon, Japan) with 409, 488, or 555 nm laser lines. Image stacks were processed by CZ software (Nikon, Japan). Adjustments of the brightness and contrast were performed using Photoshop CS4 software (Adobe Systems, Mountain View, CA, USA).

### Animal models (pulp injury repair models)

Healthy SD rats of specific pathogen-free (SPF) grade weighing 250~350 g were used in this study. Each animal was treated with various pulp capping materials on the first molar of the ABCD region, and the animals were grouped as follows. Blank control: 3 SD rats with no surgery. Negative control: 6 SD rats with saline solution as capping material. Mineral trioxide aggregate (MTA): 6 SD rats with MTA as capping material.

After intraperitoneal injection of chloral hydrate for anesthesia, the rat oral cavities were disinfected with 75% alcohol. A tungsten steel drill and 15# K-files were used to drill to the pulp. A gelatin sponge containing normal saline was placed in the perforation. MTA (Dentsply Maillefer, Switzerland) was mixed as recommended by the manufacturer and used to directly seal the perforation; the perforation was filled with glass ionomer cement. Acetaminophen was given for 2 days after the surgery. The rats were euthanized 30 days after the surgery, and their upper and lower jaws were acquired.

### Tissue preparation and staining

The rat jaw samples were fixed in 4% paraformaldehyde and decalcified with 0.5 mol·L^−1^ EDTA solution for 6 weeks before paraffin embedding. Tissue blocks were cut using a type 820 Spencer microtome at 5–7 µm. For histological analysis, sections were placed in an oven at 60 °C for 30 min, deparaffinized in xylene, and rehydrated in a decreasing ethanol gradient before staining with hematoxylin and eosin (H&E) or used for immunofluorescence analysis.

### Cell culture, treatment, and transfection

Human DPCs were isolated and cultured according to the previously described method.^54,55^ Cells between passages 3 and 6 were used. hDPCs were treated with 50 μg·mL^−1^ ascorbic acid (Sigma-Aldrich, St. Louis, MO, USA), 10 mmol·L^−1^ glycerophosphate (Sigma-Aldrich, St. Louis, MO, USA), and 100 nmol·L^−1^ dexamethasone (Sigma-Aldrich, St. Louis, MO, USA) for 7, 14, and 21 days.

The human Cx43-encoding gene *GJA1* (reference number: NM_000165) was overexpressed and inhibited by lentivirus transfection. The lentiviral vectors pLenti-EF1a-EGFP-P2A-Puro-CMV-GJA1-3Flag and pLKD-CMV-G&PR-U6-shRNA(GJA1) were constructed (Obio Technology (Shanghai) Corp., Ltd., China) to overexpress or inhibit *GJA1*, respectively. For transfection, the cells were seeded in a six-well plate and cultured to 30%–50% confluence. Twenty-four hours later, the cells were transfected with a virus concentration of 20 MOI in serum-free Dulbecco’s modified Eagles medium (DMEM) according to the manufacturer’s instructions. The medium was replaced with DMEM plus 10% fetal bovine serum after 8 h. The dental pulp cells were then induced to differentiate into odontoblasts as described above. Cells with stable transfection (Fig. 5c) were used in the gene silencing and overexpression experiments.

### Alizarin red staining

Cells cultured for 0, 7, and 14 days were fixed with 4% paraformaldehyde for 20 min at room temperature. Then, the cells were stained with 40 mmol·L^−1^ Alizarin red S (pH 4.2, Sigma-Aldrich, St. Louis, MO, USA) for 10 min at room temperature. After washing, the mineralized nodules were observed under an optical microscope.

### Real-time quantitative PCR analysis

Total RNA was extracted from hDPCs using TRIzol reagent according to the manufacturer’s instructions (Invitrogen Life Technologies, Carlsbad, CA, USA). cDNA was synthesized using iScript and was used for quantitative PCR with SYBR Green. The primer sequences were as follows: Cx43: 5′-TCTCGCCTATGTCTCCTCCT-3′ and 5′-TGCTCACTTGCTTGCTTGTT-3′; DSPP: 5′-GCAGTGACAGTAGCGATAGC-3′ and 5′-CTATTGCTGCTGTCGTTGCTA-3′; and ACT: 5′-TTCTACAATGAGCTGCGTG-3′ and 5′-CTCAAACATGATCTGGGTC-3′.

The reactions were performed using an ABI 7300HT apparatus.

### Western blot analysis

To analyze cellular protein levels, cells were harvested by scraping and lysed. The lysates were electrophoresed through 10% sodium dodecyl sulfate polyacrylamide gels, transferred to the membranes, and probed with antibodies according to the previously described method.^56^

### Immunofluorescence

hDPCs were plated in a 24-well plate for 24 h and fixed with 4% paraformaldehyde for 20 min before treatment as described above. The cells were viewed under a fluorescence microscope (Carl Zeiss, Göttingen, Germany).

### Statistical analysis

Each experiment was repeated at least three times. Differences were analyzed using one-way analysis of variance (SPSS 16.0, SPSS Inc., Chicago, IL, USA). The significance level was set at *P* < 0.05.

## Data Availability

All data generated or analyzed during this study are included in this published article.
